# A nomogram integrating CTPA parameters and serum biomarkers to predict 30-day mortality and refine risk stratification in non-high-risk acute pulmonary embolism

**DOI:** 10.3389/fcvm.2026.1926230

**Published:** 2026-09-09

**Authors:** Yang Bai, Yueli Dai, Jianchun Peng, Ting Xie

**Affiliations:** 1 Department of Radiology, The Second Affiliated Hospital, University of South China, Hengyang, China; 2 Hengyang Medical School, University of South China, Hengyang, China; 3 Emergency Medicine Center, The Second Affiliated Hospital, University of South China, Hengyang, China

**Keywords:** 30-day all-cause mortality, biomarker, CT pulmonary angiography, non-high-risk acute pulmonary embolism, prediction model, risk stratification

## Abstract

**Objective:**

To explore the predictive value of CT pulmonary angiography (CTPA) imaging parameters combined with serum biomarkers for 30-d all-cause mortality in non-high-risk acute pulmonary embolism (APE), and to compare parameters between simplified Pulmonary Embolism Severity Index (sPESI)-based risk strata.

**Methods:**

This retrospective study enrolled 250 first-episode non-high-risk APE patients (50 deaths, 200 survivors). CTPA-derived pulmonary artery obstruction index (PAOI), right/left ventricular diameter ratio (RV/LV), main pulmonary/ascending aorta ratio, and serum biomarkers high-sensitivity cardiac troponin I (hs-cTnI), brain natriuretic peptide (BNP), D-dimer, and blood lactate (BLa) were collected. Independent risk factors were identified using Firth penalized likelihood logistic regression to construct a nomogram, which was internally validated. The incremental predictive value over sPESI was quantified. Cox regression was used to assess prognostic values and interactions. Spearman correlation analysis was conducted for relationships between imaging and biomarkers. Patients were stratified by sPESI into intermediate-risk (≥ 1 point) and low-risk (0 points) groups for comparative and survival analyses.

**Results:**

Death *vs.* survival groups differed significantly in syncope, age >80 years, SBP <100 mmHg, PAOI, RV/LV, hs-cTnI, BNP, and BLa (*P* < 0.05). Independent predictors were syncope (*OR* = 5.846, 95% *CI*: 1.552–22.030), SBP <100 mmHg (*OR* = 6.118, 95% *CI*: 1.899–19.704), RV/LV (*OR* = 1.285, 95% *CI*: 1.028–1.607), BNP (*OR* = 1.002, 95% *CI*: 1.000–1.004), and BLa (*OR* = 2.162, 95% *CI*: 1.372–3.406). The nomogram showed good discrimination (AUC = 0.878), calibration, and clinical net benefit, significantly outperforming sPESI (AUC = 0.713, *P* < 0.001). Cox regression confirmed several predictors but sPESI was not an independent factor (*HR* = 0.478, *P* = 0.178). PAOI and RV/LV correlated positively with hs-cTnI, BNP, and BLa (*r* = 0.345–0.427, *P* < 0.001). Intermediate-risk patients had higher mortality, PAOI, RV/LV, BNP, and BLa than low-risk patients (*P* < 0.05).

**Conclusion:**

The nomogram incorporating syncope, SBP <100 mmHg, RV/LV, BNP, and BLa, is an effective tool for predicting 30-day mortality in non-high-risk APE patients, demonstrating superior performance over sPESI. Combining CTPA imaging and serum biomarkers offers independent and complementary prognostic information, enhancing risk stratification.

## 1 Introduction

Acute pulmonary embolism (APE) is an acute pulmonary circulatory disorder syndrome caused by embolic occlusion of the pulmonary artery and its branches. It ranks third among cardiovascular emergencies, following only coronary heart disease and stroke (1). Based on hemodynamic status, APE is classified as high-risk or non-high-risk, with non-high-risk patients comprising the majority of diagnosed cases. Despite advances in clinical management and the widespread adoption of standardized anticoagulation therapy that have improved overall prognosis, the 30-day all-cause mortality rate among non-high-risk patients remains 3.5% (2), indicating that the risk of short-term adverse outcomes should not be underestimated.

Current guidelines recommend early risk stratification based on hemodynamic status and the simplified Pulmonary Embolism Severity Index (sPESI). However, the discriminative performance of sPESI is limited. Large-scale external validation from the CURES revealed that the area under the curve (AUC) of the sPESI score for predicting 30-day all-cause mortality in non-high-risk APE was only 0.712, and the AUC of sPESI ≥1 point was 0.669 (2). This limited performance may be attributable to the fact that sPESI incorporates only bedside parameters such as age, vital signs, and comorbidities, without including objective indicators of right ventricular function, thrombus burden, or tissue hypoperfusion. Consequently, substantial prognostic heterogeneity persists within both intermediate- and low-risk strata, with some sPESI-defined low-risk patients still experiencing early fatal events (3, 4). Therefore, developing more precise individualized risk assessment tools is of considerable clinical importance.

Computed tomography pulmonary angiography (CTPA) enables simultaneous evaluation of thrombus burden via the pulmonary artery obstruction index (PAOI), right ventricular function via the right/left ventricular diameter ratio (RV/LV), and pulmonary arterial pressure loading via the main pulmonary artery/ascending aorta diameter ratio (PA/AO). Among serum biomarkers, high-sensitivity cardiac troponin I (hs-cTnI) reflects myocardial microinjury, brain natriuretic peptide (BNP) reflects ventricular wall stretch, D-dimer reflects coagulation and fibrinolysis activation, and blood lactate (BLa) reflects tissue hypoperfusion, all of which are closely associated with adverse prognosis in APE (5–8). Nevertheless, most existing research has investigated the prognostic value of individual imaging parameters or serum biomarkers in isolation. Systematic investigations that integrate the aforementioned CTPA parameters with serum biomarkers and employ Firth penalized likelihood regression, appropriate for rare-event data, to construct mortality prediction models for non-high-risk APE patients remain scarce.

To address this gap, the present study combined CTPA imaging parameters with serum biomarkers to construct and internally validate a nomogram for predicting 30-day all-cause mortality in non-high-risk APE patients using Firth penalized likelihood logistic regression. We also quantified its incremental value over the sPESI score. Furthermore, through subgroup analyses and interaction tests, we aimed to deeply explore the relationship between sPESI strata and objective indices, thereby providing evidence for precise risk stratification in this population.

## 2 Materials and methods

### 2.1 Sample size calculation

The 30-day all-cause mortality rate in APE patients decreased from 6.6% (2001–2005) to 4.9% (2010–2013) in the large international RIETE registry (*n* = 23,858) (9), and was 3.5% (*n* = 6,873) in the prospective CURES cohort of non-high-risk APE patients (2). Thus, 30-day all-cause mortality in non-high-risk APE represents a relatively rare event. A conventional prospective cohort design would require an extremely large sample size to accrue a sufficient number of positive events (10). Therefore, this study adopted a retrospective 1:4 case-control design to enhance statistical efficiency. A total of 250 patients with first-episode non-high-risk APE were included, with 50 in the 30-day all-cause death group and 200 in the survival group. In multivariable regression analysis, although the rule of thumb suggests that at least 5 to 9 events per variable are required for logistic regression (11), rare-event data frequently lead to small-sample bias or complete separation issues with conventional maximum likelihood estimation. To address this, the present study employed Firth penalized likelihood logistic regression for independent risk factor selection and prediction model construction. This method applies a Jeffreys prior penalty to the likelihood function, effectively eliminating first-order asymptotic bias and providing stable parameter estimates and reliable hypothesis tests even when the number of positive events is relatively small (12, 13).

### 2.2 Patient data and grouping

This retrospective study enrolled 250 patients with first-episode non-high-risk APE at our hospital between April 2022 and July 2024, including cases diagnosed at admission and during hospitalization. Inclusion criteria included: (1) intermediate- or low-risk APE diagnosed according to the 2019 ESC/ERS Guidelines for the Diagnosis and Management of APE (sPESI criteria) (14); (2) age ≥18 years; (3) symptom onset ≤14 days; (4) CTPA and serum biomarker measurements completed within 24 h of admission; (5) complete clinical data, encompassing demographic features, medical history, laboratory findings, treatment regimens, and follow-up outcomes; (6) complete 30-day survival outcomes obtainable via the hospital electronic medical record system and/or telephone follow-up; (7) approval obtained from the hospital's Ethics Committee, with informed consent waived because of the retrospective nature of this study. Exclusion criteria were: (1) prior history of pulmonary embolism; (2) receipt of specific therapy for pulmonary embolism, such as thrombolysis or anticoagulation, prior to admission; (3) chronic thromboembolic pulmonary hypertension or chronic pulmonary embolism; (4) pregnancy or lactation; (5) severe infection or sepsis meeting the Sepsis-3 diagnostic criteria; (6) severe hepatic or renal insufficiency or receipt of renal replacement therapy; (7) end-stage malignancy or receipt of palliative care; (8) active bleeding or platelet count <30 × 10^9^/L; (9) discharge against medical advice or transfer to a non-affiliated institution within 48 h; (10) severe valvular heart disease (moderate or severe aortic/mitral stenosis or regurgitation) or congenital heart disease; (11) high-risk pulmonary embolism defined by shock [systolic blood pressure (SBP) <90 mmHg with end-organ hypoperfusion] or persistent hypotension (SBP <90 mmHg or a decrease ≥40 mmHg for >15 min) at admission (14). All-cause death within 30 days of admission was the primary endpoint. The participants were allocated to the death group (*n* = 50) or the survival group (*n* = 200).

### 2.3 Collection of clinical presentations and baseline characteristics

(1) Clinical presentations: The primary symptoms and signs at admission were recorded, encompassing fever (≥37.3°C), cough, hemoptysis, syncope, dyspnea, pleuritic chest pain, substernal chest pain, unilateral limb swelling, and unilateral limb pain. Each parameter was classified as “present” or “absent” based on medical record documentation.
(2) Demographic features: Age, sex, and obesity [body mass index (BMI) ≥28.0 kg/m^2^].
(3) Comorbidity history: Hypertension, diabetes mellitus, history of stroke or transient ischemic attack (TIA), history of venous thromboembolism (VTE), chronic heart failure and/or chronic pulmonary disease (CHF/CPD), malignancy, recent major bleeding (within 30 days), and recent surgery or immobilization (within 30 days).
(4) Vital signs at admission: Pulse rate ≥110 beats per min, SBP ≤ 100 mmHg, respiratory rate (RR) >30 breaths per min, and oxygen saturation (SpO_2_) <90%.
(5) CTPA imaging parameters: ① PAOI: The PAOI was calculated using the Qanadli scoring method (15). The pulmonary vascular tree was divided into 20 segments (3 segments in the right upper lobe, 2 in the middle lobe, 5 in the lower lobe; 3 in the left upper lobe, 5 in the lower lobe, and 2 in the lingula). The degree of embolic occlusion in each segment was assessed on axial CTPA images: no embolus = 0 points, partial occlusion = 1 point, and complete occlusion = 2 points. PAOI was calculated as PAOI = [∑(n × d)/40] × 100%, where *n* is the number of segments affected by embolus and d represents the degree of occlusion in that segment. PAOI range was 0%–100%, with higher values indicative of greater thrombus burden. ② RV/LV: The ratio of the maximal short-axis diameter of the right ventricle to that of the left ventricle was measured in the four-chamber view. ③ PA/AO: The maximal diameters of the main pulmonary artery and the ascending aorta were measured at the level of the pulmonary artery bifurcation on axial CTPA images, and the PA/AO was calculated.
(6) Serum biomarkers: Peripheral venous blood was collected within 24 h of admission for measurements: ① hs-cTnI: measured using a fully automated chemiluminescence immunoassay analyzer (TESMI i100, Tellgen Corporation, Shanghai, China) with the hs-cTnI assay kit (chemiluminescent immunoassay). The normal reference range was <0.04 ng/mL. ② BNP: measured using an electrochemiluminescence analyzer (cobas e411, Roche Diagnostics, Shanghai, China) with the B-type natriuretic peptide assay kit (electrochemiluminescence method). The normal reference range was <100 pg/mL. ③ D-dimer: measured using a fully automated coagulation analyzer (MDC3500, Strong Biotechnologies, Beijing, China) with the D-dimer assay kit (immunoturbidimetric method). The conventional normal cutoff was <0.5 mg/L; for patients aged >50 years, the age-adjusted cutoff (age × 0.01 mg/L) was applied. ④ BLa: measured using a fully automated blood gas analyzer (GEM Premier 5,000, Werfen, Beijing, China) with the GEM Premier 5,000 reagent kit (normal reference range: 0.5–2.2 mmol/L).

### 2.4 APE risk stratification criteria (14)

**Figure 1:**
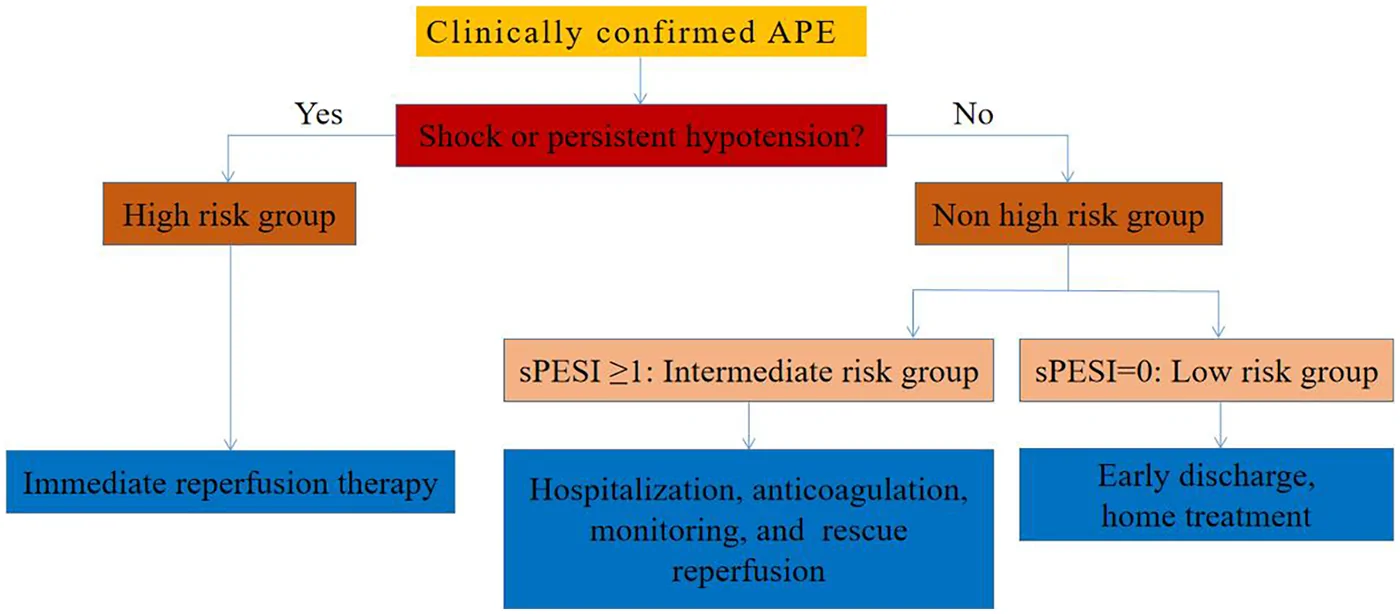
Flowchart of APE risk stratification and treatment strategy. APE, acute pulmonary embolism; sPESI, simplified Pulmonary Embolism Severity Index.

(1) High-risk definition: The presence of either of the following at admission was defined as high-risk: ① shock (SBP <90 mmHg with end-organ hypoperfusion); ② persistent hypotension (SBP <90 mmHg or a decrease ≥40 mmHg for >15 min). Patients meeting high-risk criteria were excluded.
(2) sPESI stratification for non-high-risk patients: Patients who did not meet the high-risk criteria were further stratified using the sPESI. The sPESI comprises six items: age >80 years, malignancy, CHF/CPD, pulse rate ≥110 bpm, SBP <100 mmHg, and arterial SpO_2_ < 90%, with each item scored 1 point (total score: 0–6 points). Patients were classified into intermediate-risk (≥1 point) or low-risk (0 points) group based on total score. Mortality rates, CTPA imaging parameters, and serum biomarker levels were compared between risk strata. A detailed flowchart of APE risk stratification and treatment strategy is presented in Figure 1.

### 2.5 Statistical methods

All analyses were performed employing SPSS 27.0 and R 4.5.1. Normality of continuous variables was tested utilizing the Shapiro–Wilk test. Non-normally distributed data were expressed as M (P_25_, P_75_) and compared between groups applying the Mann–Whitney *U*-test. Normally distributed data were described as $\bar{x}$  ± *s*; Levene's test was utilized to assess homogeneity of variances, and between-group comparisons were carried out with the independent-samples *t*-test (equal variances assumed) or Welch's *t*-test (equal variances not assumed). Categorical data were reported as *n* (%) and compared by the *χ*^2^ test (expected count ≥5) or Fisher's exact test (expected count <5). Firth penalized likelihood logistic regression was applied to screen independent risk factors; variables with *P* < 0.05 in univariate analysis were entered simultaneously into the multivariable model, calculating adjusted odds ratios (*OR*s) with 95% confidence intervals (*CI*s). A nomogram prediction model was built based on independent predictors with statistical significance. Receiver operating characteristic (ROC) curves were generated to assess the discrimination of the model and the sPESI score. Stratified bootstrap internal validation with 500 resamples was used to obtain the optimism-corrected concordance index (C-index) with 95% *CI*. Calibration assessment was achieved through calibration plots and the Hosmer-Lemeshow test. Decision curve analysis (DCA) was performed to quantify the net benefit of using the model to guide treatment across different risk thresholds. The continuous net reclassification improvement (NRI) and integrated discrimination improvement (IDI) were calculated to quantify the incremental predictive value of the nomogram over the sPESI score. Univariate and multivariate Cox proportional hazards regression models were employed to assess prognostic factors for 30-day all-cause mortality, providing hazard ratios (*HR*) and 95% *CI*s. Interactions between sPESI strata and RV/LV or BLa were tested. Spearman correlation analysis was conducted for CTPA imaging parameter-serum biomarker correlations. Survival curves were generated using the Kaplan–Meier method, and intergroup differences were compared using the log-rank test. *P* < 0.05 was considered statistically significant.

## 3 Results

### 3.1 Comparison of clinical presentations between the death and survival groups

The incidence of syncope in the death group was markedly higher than the survival group (16.00% *vs.* 3.50%, *P* < 0.05). No significant between-group differences were observed in fever, cough, hemoptysis, dyspnea, chest pain, or limb pain/swelling (all *P* > 0.05; Table 1).

**Table 1:** Comparison of clinical presentations between the death and survival groups [n (%)].

Clinical presentation	Total (*n* = 250)	Death group (*n* = 50)	Survival group (*n* = 200)	*χ* ^2^	*P*
Fever	25 (10.00)	7 (14.00)	18 (9.00)	1.111	0.292
Cough	61 (24.40)	11 (22.00)	50 (25.00)	0.195	0.659
Hemoptysis	18 (7.20)	4 (8.00)	14 (7.00)	^a^	0.764
Syncope	17 (6.80)	10 (20.00)	7 (3.50)	^a^	<0.001
Dyspnea	193 (77.20)	42 (84.00)	151 (75.50)	1.642	0.200
Pleuritic chest pain	100 (40.00)	18 (36.00)	82 (41.00)	0.417	0.519
Substernal chest pain	36 (14.40)	11 (22.00)	25 (12.50)	2.929	0.087
Unilateral limb swelling	61 (24.40)	7 (14.00)	54 (27.00)	3.665	0.056
Unilateral limb pain	14 (5.60)	3 (6.00)	11 (5.50)	^a^	1.000

a Fisher's exact test.

### 3.2 Comparison of baseline characteristics between the death and survival groups

Significant between-group differences were detected in age >80 years, SBP <100 mmHg, PAOI, RV/LV, hs-cTnI, BNP, and BLa (all *P* < 0.05). No significant between-group differences were revealed in sex, obesity, hypertension, diabetes mellitus, stroke/TIA history, VTE history, CHF/CPD, malignancy, recent major bleeding, recent surgery/immobilization, pulse rate ≥110 bpm, RR > 30 bpm, arterial SpO_2_ < 90%, PA/AO, or D-dimer (all *P* > 0.05; Table 2).

**Table 2:** Comparison of baseline characteristics between the death and survival groups.

Variable	Death group (*n* = 50)	Survival group (*n* = 200)	*χ* ^2^ */Z/t*	*P*
Age >80 years	10 (20.00)	12 (6.00)	^a^	0.004
Male/female	24 (48.00)	106 (53.00)	0.401	0.527
Obesity	10 (20.00)	47 (23.50)	0.278	0.598
Hypertension	21 (42.00)	76 (38.00)	0.270	0.604
Diabetes mellitus	9 (18.00)	30 (15.00)	0.273	0.601
Stroke/TIA history	7 (14.00)	18 (9.00)	1.111	0.292
VTE history	5 (10.00)	14 (7.00)	^a^	0.549
CHF/CPD	9 (18.00)	26 (13.00)	0.831	0.362
Malignancy	3 (6.00)	8 (4.00)	^a^	0.464
Recent major bleeding	2 (4.00)	1 (0.50)	^a^	0.103
Recent surgery/immobilization	9 (18.00)	42 (21.00)	0.222	0.638
Pulse ≥110 bpm	17 (34.00)	43 (21.50)	3.427	0.064
SBP <100 mmHg	11 (22.00)	10 (5.00)	^a^	<0.001
RR >30 bpm	8 (16.00)	17 (8.50)	2.500	0.114
Arterial SpO_2_ < 90%	7 (14.00)	12 (6.00)	^a^	0.072
PAOI	39.00 (27.00, 50.00)	31.00 (20.50, 41.50)	3.616	<0.001
RV/LV	1.28 ± 0.31	1.01 ± 0.18	6.143	<0.001
PA/AO	0.92 ± 0.16	0.88 ± 0.14	1.537	0.126
hs-cTnI (ng/mL)	0.54 (0.19, 0.77)	0.24 (0.11, 0.39)	4.178	<0.001
BNP (pg/mL)	552.50 (313.00, 801.00)	286.00 (184.50, 414.50)	5.496	<0.001
D-dimer (mg/L)	3.72 (1.68, 5.39)	3.02 (1.57, 4.67)	1.772	0.076
BLa (mmol/L)	2.84 (1.67, 3.96)	1.58 (1.11, 2.15)	6.181	<0.001

a Fisher's exact test.

TIA, transient ischemic attack; VTE, venous thromboembolism; CHF/CPD, chronic heart failure/chronic pulmonary disease; SBP, systolic blood pressure; RR, respiratory rate; SpO_2_, peripheral oxygen saturation; PAOI, pulmonary artery obstruction index; RV/LV, right/left ventricular diameter ratio; PA/AO, main pulmonary artery/ascending aorta diameter ratio; hs-cTnI, high-sensitivity cardiac troponin I; BNP, brain natriuretic peptide; BLa, blood lactate.

### 3.3 Firth regression analysis of 30-day all-cause mortality in non-high-risk APE patients

**Figure 2:**
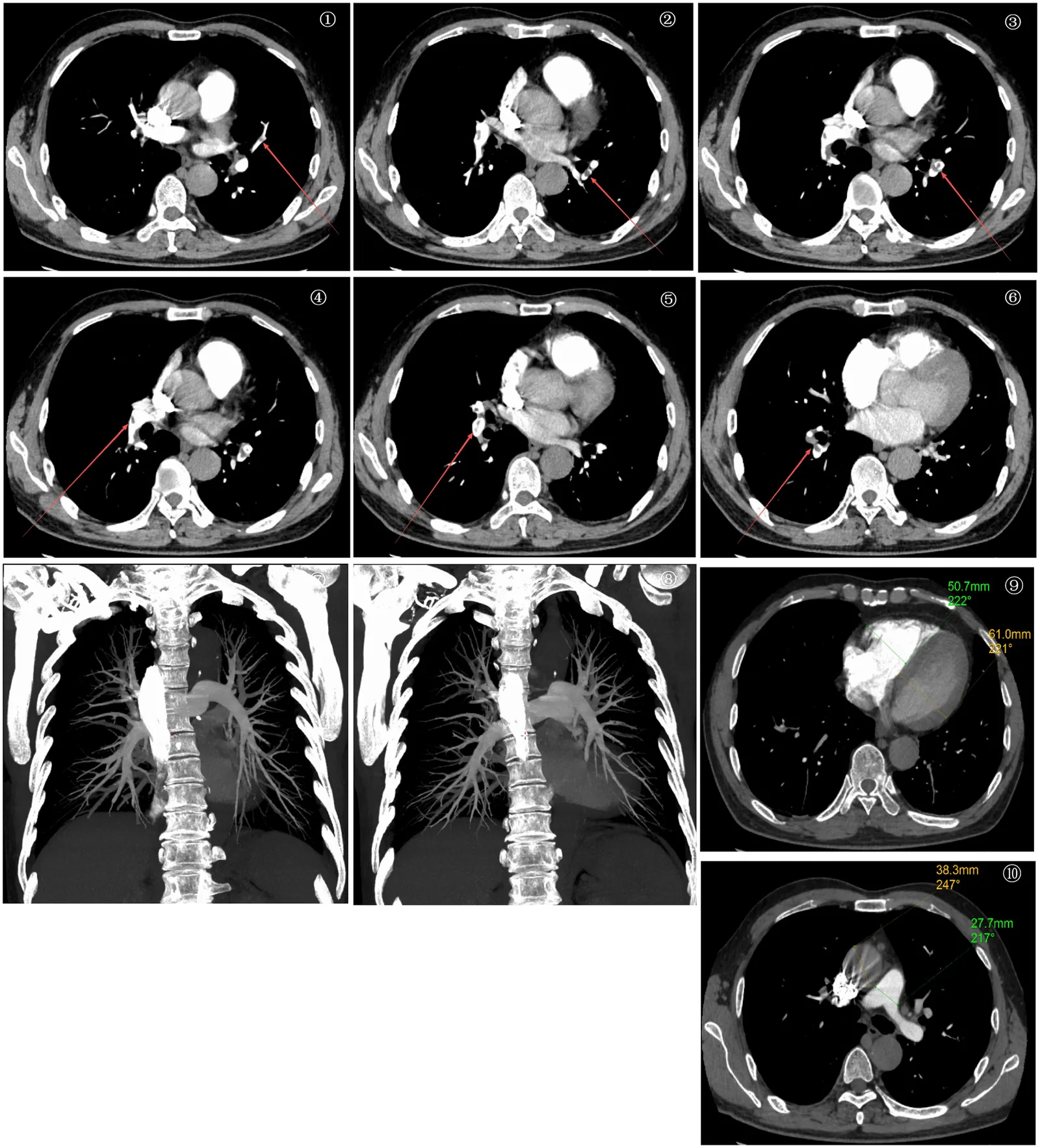
CTPA and MIP images of a representative surviving APE patient. Male patient, 63 years old, with acute lower extremity deep vein thrombosis secondary to APE, included in the survival group. Panels ①–⑥: axial dual-energy CTPA images. Panels ⑦–⑧: coronal MIP images showing no widening of the main pulmonary artery trunk (diameter smaller than the aorta at the same level), with low-attenuation filling defects visible in the left upper lobe apicoposterior segment, some basal segments of the left lower lobe, the right upper lobe posterior, apical, and anterior segments, the right middle lobe lateral segment, and the right lower lobe basal segment pulmonary arteries (1 segment in the left upper lobe, partial occlusion; 4 segments in the left lower lobe, partial occlusion; 3 segments in the right upper lobe, partial occlusion; 1 segment in the right middle lobe, partial occlusion; 2 segments in the right lower lobe, partial occlusion), yielding a calculated PAOI of 27.5%. Panel ⑨: measurement of the maximal short-axis diameters of the right and left ventricles in the four-chamber view, with an RV/LV of 0.83. Panel ⑩: measurement of the maximal diameters of the main pulmonary artery and ascending aorta at the pulmonary artery bifurcation level, with a PA/AO of 0.72. CTPA, computed tomography pulmonary angiography; MIP, maximum intensity projection; APE, acute pulmonary embolism; PAOI, pulmonary artery obstruction index; RV/LV, right/left ventricular diameter ratio; PA/AO, main pulmonary artery/ascending aorta diameter ratio.

**Figure 3:**
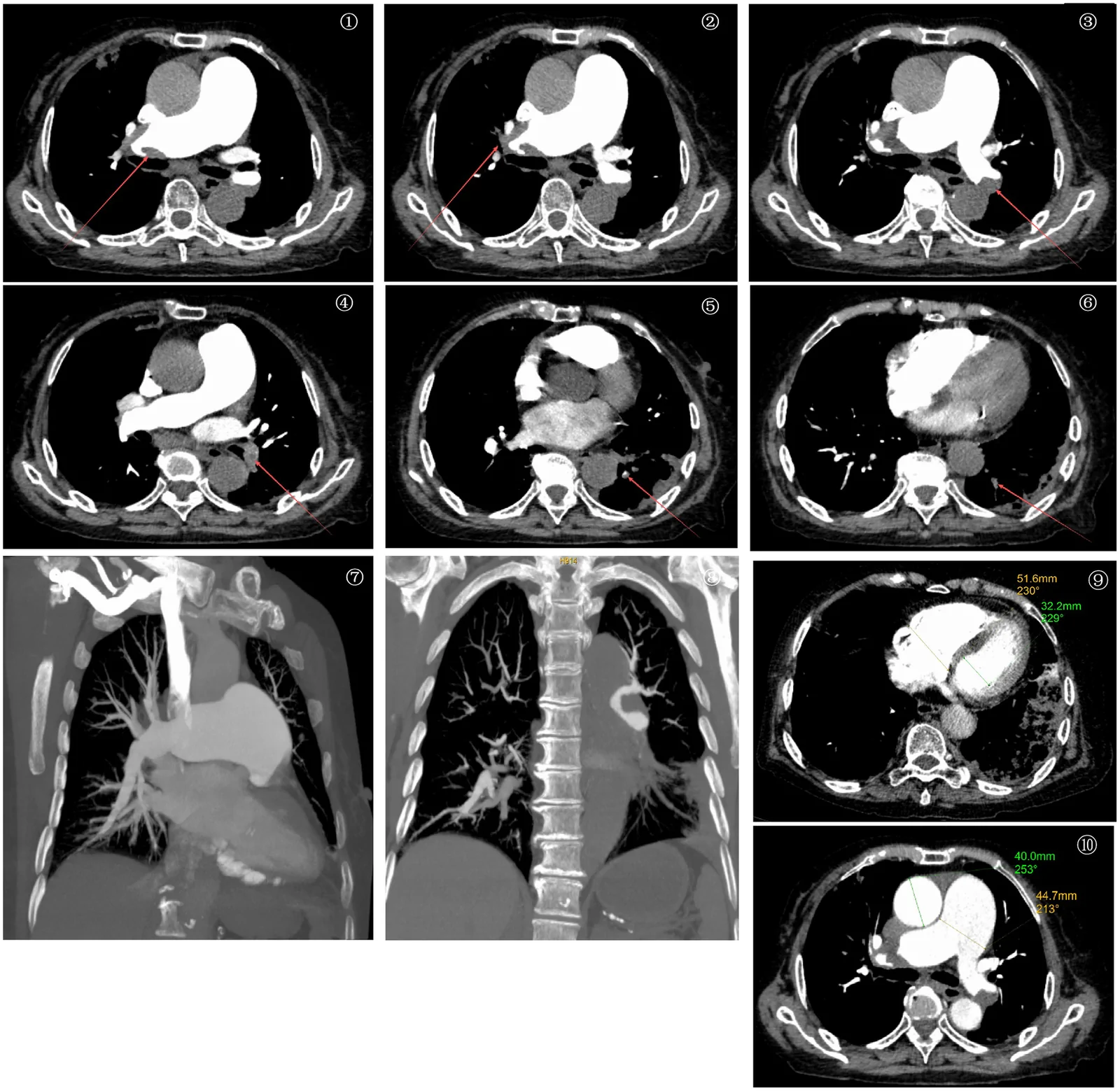
CTPA and MIP images of a representative deceased APE patient. Female patient, 85 years old, hospitalized with APE, included in the death group. Panels ①–⑥: axial dual-energy CTPA images. Panels ⑦–⑧: coronal MIP images showing widening of the main pulmonary artery trunk (diameter larger than the aorta at the same level), with multiple low-attenuation non-enhancing filling defects in the left and right main pulmonary arteries, left upper and lower lobe pulmonary arteries, and right middle and lower lobe pulmonary arteries (2 segments in the left upper lobe, partial occlusion; 5 segments in the left lower lobe, complete occlusion; 1 segment in the right middle lobe, complete occlusion; 3 segments in the right lower lobe, partial occlusion), yielding a calculated PAOI of 42.5%. Panel ⑨: RV/LV of approximately 1.60. Panel ⑩: PA/AO of 1.18. CTPA, computed tomography pulmonary angiography; MIP, maximum intensity projection; APE, acute pulmonary embolism; PAOI, pulmonary artery obstruction index; RV/LV, right/left ventricular diameter ratio; PA/AO, main pulmonary artery/ascending aorta diameter ratio.

Variables (*P* < 0.05 in univariate analysis) were simultaneously entered into the Firth penalized likelihood logistic regression model. Variable assignments are presented in Table 3. Syncope (*OR* = 5.846, 95% *CI*: 1.552–22.030), SBP <100 mmHg (*OR* = 6.118, 95% *CI*: 1.899–19.704), per 0.1-unit increase in RV/LV (*OR* = 1.285, 95% *CI*: 1.028–1.607), elevated BNP (*OR* = 1.002, 95% *CI*: 1.000–1.004), and elevated BLa (*OR* = 2.162, 95% *CI*: 1.372–3.406) were independent predictors of 30-day all-cause mortality in non-high-risk APE patients (all *P* < 0.05). Age >80 years, PAOI, and hs-cTnI did not reach statistical significance (all *P* > 0.05; Table 4). Representative dual-energy CTPA axial and coronal maximum intensity projection (MIP) images from a surviving patient and a deceased patient before treatment are shown in Figures 2, 3, respectively.

**Table 3:** Variable assignment table.

Variable	Assignment
Syncope	No = 0, Yes = 1
Age >80 years	No = 0, Yes = 1
SBP <100 mmHg	No = 0, Yes = 1
PAOI	Continuous
RV/LV	Continuous
hs-cTnI	Continuous
BNP	Continuous
BLa	Continuous

SBP, systolic blood pressure; PAOI, pulmonary artery obstruction index; RV/LV, right/left ventricular diameter ratio; hs-cTnI, high-sensitivity cardiac troponin I; BNP, brain natriuretic peptide; BLa, blood lactate.

**Table 4:** Firth regression analysis of 30-day all-cause mortality in non-high-risk APE patients.

Variable	*β*	*SE*	*Z*	*P*	*OR* (95% *CI*)
Syncope	1.766	0.677	2.609	0.009	5.846 (1.552–22.030)
Age >80 years	1.141	0.607	1.879	0.060	3.129 (0.952–10.282)
SBP <100 mmHg	1.811	0.597	3.035	0.002	6.118 (1.899–19.704)
PAOI	−0.017	0.017	−1.020	0.308	0.983 (0.951–1.016)
RV/LV	0.251	0.114	2.198	0.028	1.285 (1.028–1.607)
hs-cTnI	0.968	0.632	1.533	0.125	2.634 (0.764–9.084)
BNP	0.002	0.001	1.997	0.046	1.002 (1.000–1.004)
BLa	0.771	0.232	3.324	0.001	2.162 (1.372–3.406)

RV/LV was entered into the model as the original continuous variable divided by 0.1, reflecting the risk change per 0.1-unit increment. APE, acute pulmonary embolism; SBP, systolic blood pressure; PAOI, pulmonary artery obstruction index; RV/LV, right/left ventricular diameter ratio; hs-cTnI, high-sensitivity cardiac troponin I; BNP, brain natriuretic peptide; BLa, blood lactate; SE, standard error; OR, odds ratio; CI, confidence interval.

### 3.4 Nomogram construction, evaluation, and incremental value

**Figure 4:**
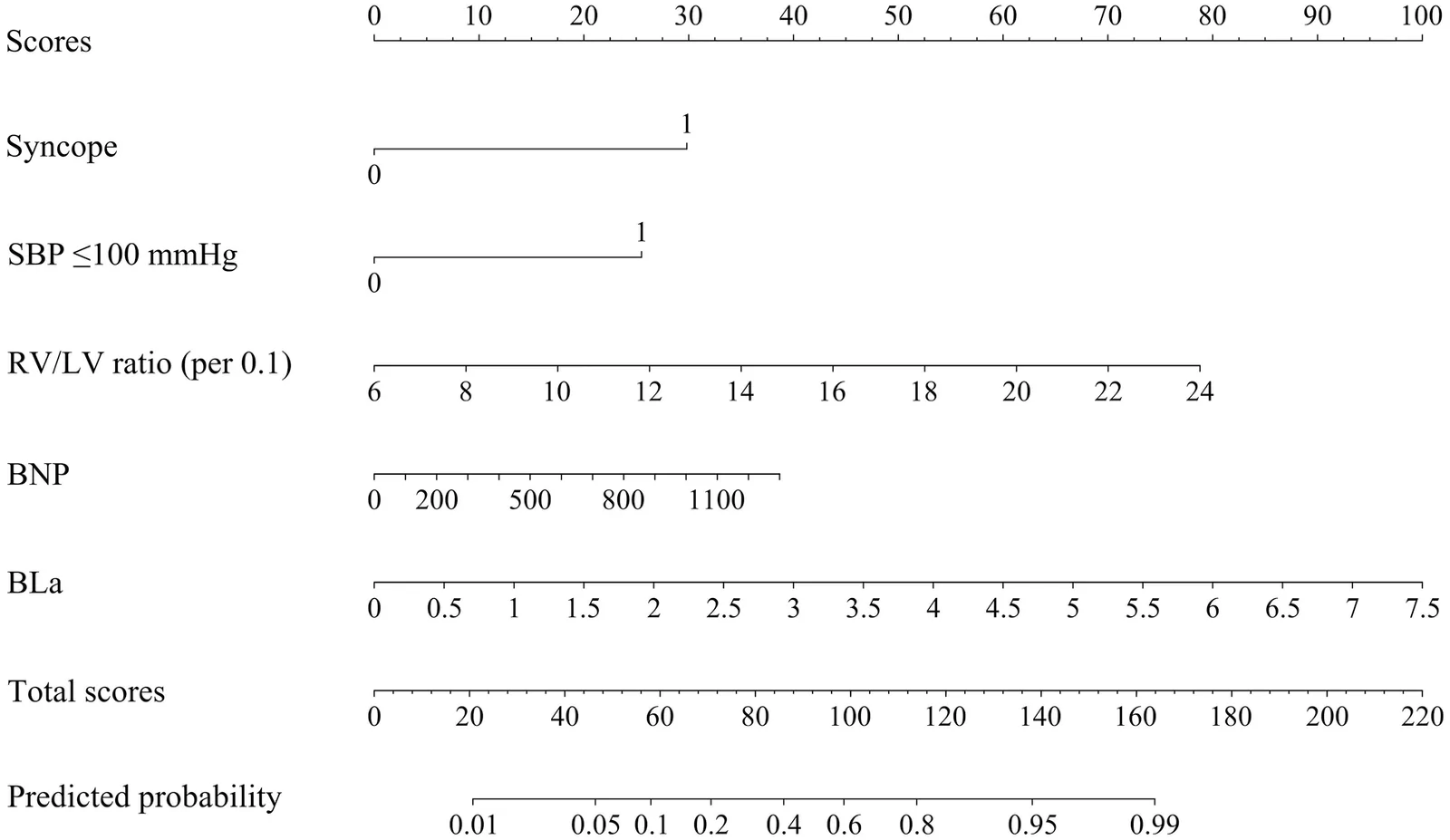
Nomogram prediction model. SBP, systolic blood pressure; RV/LV, right/left ventricular diameter ratio; BNP, brain natriuretic peptide.

**Figure 5:**
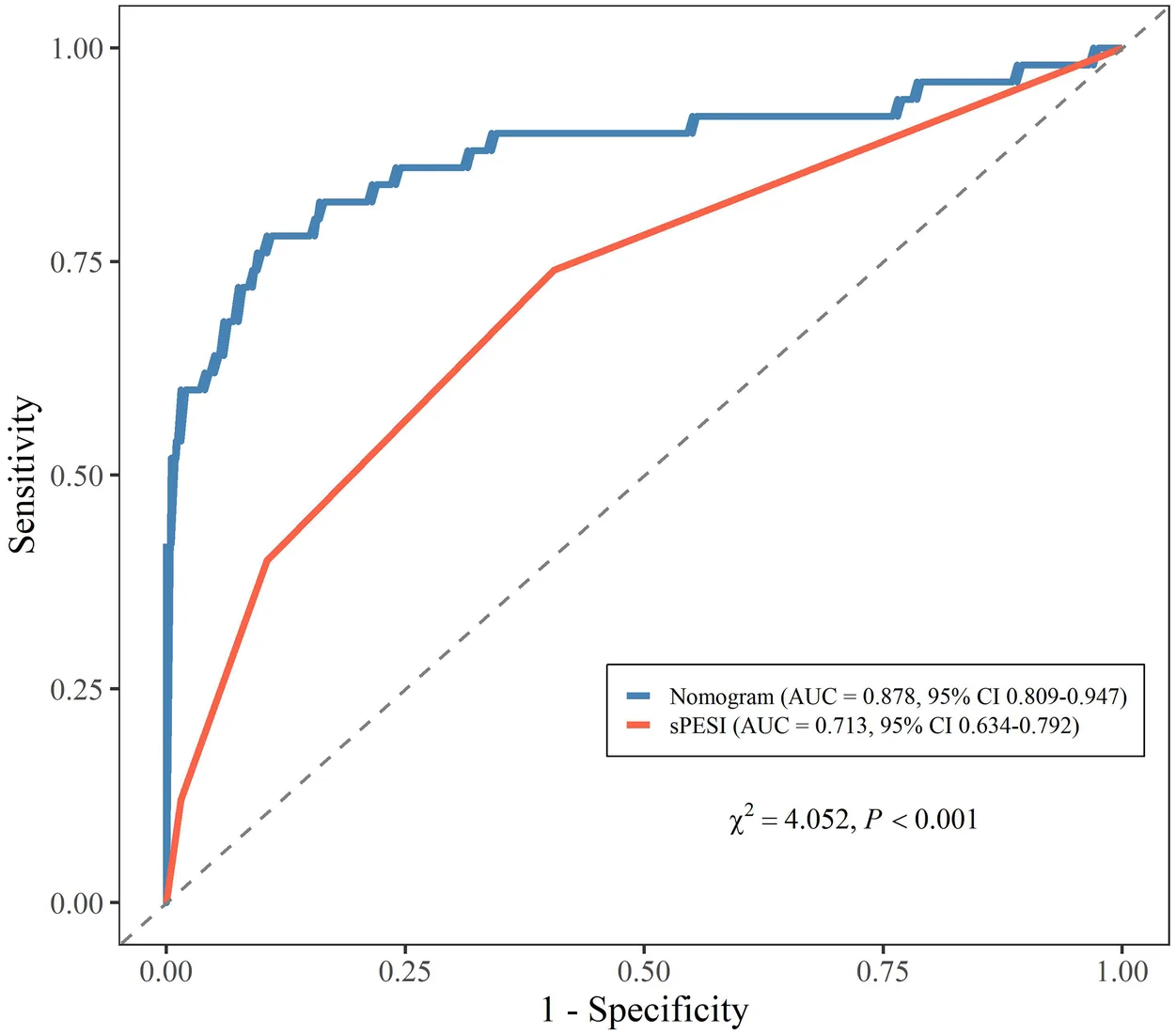
Combined ROC curves of the nomogram and the sPESI score. ROC, receiver operating characteristic; sPESI, simplified Pulmonary Embolism Severity Index; AUC, area under the curve; CI, confidence interval.

**Figure 6:**
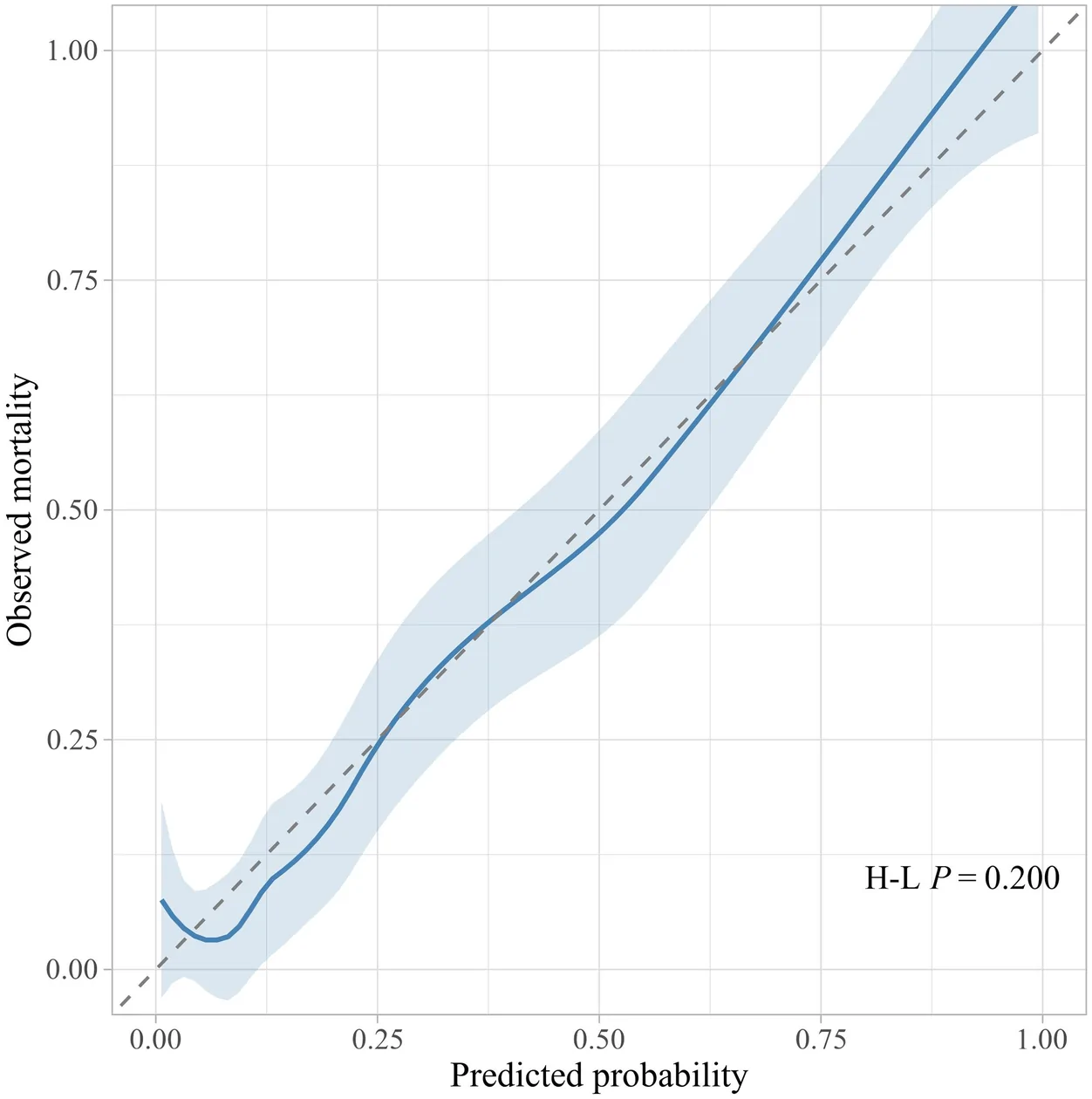
Calibration plot of the prediction model.

**Figure 7:**
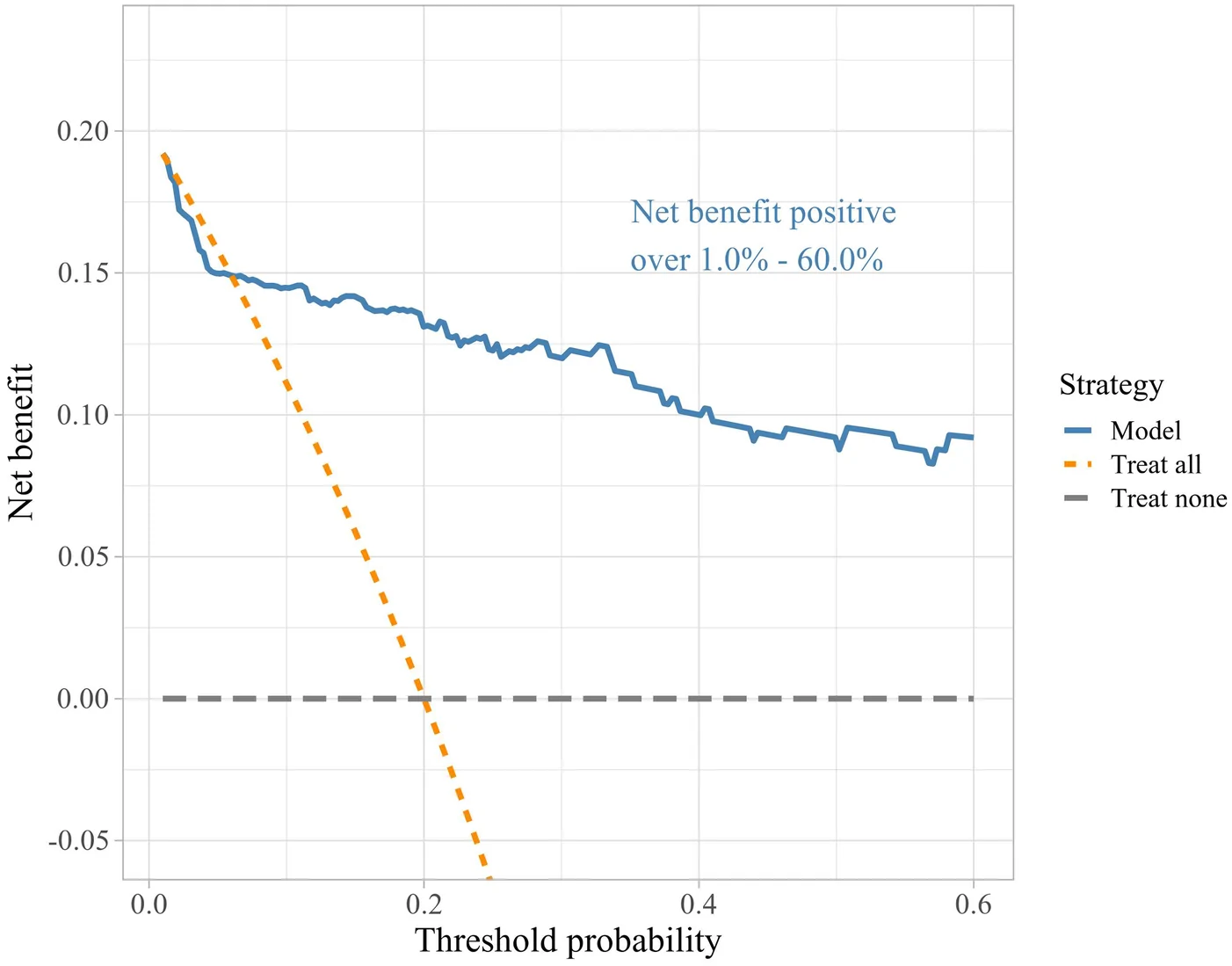
Decision curve analysis of the prediction model.

A nomogram for predicting 30-day all-cause mortality in non-high-risk APE patients was constructed using the statistically significant variables in the multivariable Firth regression model (Figure 4). The combined ROC curve for the nomogram and the sPESI score (Figure 5) showed that the nomogram achieved an AUC of 0.878 (95% *CI*: 0.809–0.947) for predicting 30-day all-cause mortality. After optimism correction using 500 stratified bootstrap resamples, the corrected C-index was 0.858 (95% *CI*: 0.800–0.926), implying good discrimination. At the optimal threshold of 0.248, the corresponding maximum Youden index was 0.675 (sensitivity, 78.00%; specificity, 89.50%). The sPESI score had an AUC of 0.713 (95% *CI*: 0.634–0.792), with a maximum Youden index of 0.335, a sensitivity of 74.00%, and a specificity of 59.50%. The nomogram's AUC was significantly superior to the sPESI score (DeLong *χ*^2^ = 19.391, *P* < 0.001). The calibration plot displayed good agreement between predicted and observed probabilities (Hosmer-Lemeshow test: *χ*^2^ = 11.026, *P* = 0.200), suggesting satisfactory calibration (Figure 6). The net benefit of applying the prediction model to guide clinical intervention exceeded that of the “treat all” or “treat none” strategies across threshold probabilities of 1%–60% (Figure 7). The continuous NRI was 0.465 (95% *CI*: 0.299–0.640, *P* < 0.001) and IDI was 0.344 (95% *CI*: 0.244–0.440, *P* < 0.001), confirming the nomogram's significant improvement in risk reclassification and overall discrimination over the sPESI score.

### 3.5 Univariate and multivariate Cox regression analyses

Univariate Cox regression (Table 5) identified 13 variables (syncope, age >80 years, recent major bleeding, pulse ≥110 bpm, SBP <100 mmHg, SpO_2_ < 90%, PAOI, RV/LV, hs-cTnI, BNP, D-dimer, BLa, sPESI strata) associated with 30-day all-cause mortality (all *P* < 0.05). Multivariate Cox regression (Table 5) revealed syncope, age >80 years, pulse ≥110 bpm, SBP <100 mmHg, hs-cTnI, D-dimer, and BLa as independent risk factors for 30-day all-cause mortality. The sPESI strata were not significant after adjustment (*HR* = 0.478, 95% *CI*: 0.163–1.399, *P* = 0.178). The multivariate model's C-index was 0.864, indicating good discrimination. Interaction analysis showed no significant interaction between sPESI strata and RV/LV (*HR* = 1.158, 95% *CI*: 0.867–1.547, *P* = 0.321) or BLa (*HR* = 0.712, 95% *CI*: 0.373–1.359, *P* = 0.303).

**Table 5:** Univariate and multivariate Cox regression for 30-day all-cause mortality.

Variable	Univariate Cox regression		Multivariate Cox regression	
	*HR* (95% *CI*)	*P*	*HR* (95% *CI*)	*P*
Syncope	4.918 (2.454–9.856)	<0.001	4.627 (2.021–10.595)	<0.001
Age >80 years	3.596 (1.796–7.199)	<0.001	7.740 (2.881–20.796)	<0.001
Recent major bleeding	5.190 (1.260–21.387)	0.023	1.320 (0.213–8.166)	0.765
Pulse ≥110 bpm	1.828 (1.018–3.282)	0.043	2.123 (1.033–4.364)	0.041
SBP <100 mmHg	4.586 (2.344–8.973)	<0.001	9.477 (3.663–24.518)	<0.001
SpO_2_ < 90%	2.351 (1.057–5.227)	0.036	2.624 (0.996–6.907)	0.051
PAOI	1.039 (1.021–1.058)	<0.001	1.004 (0.984–1.025)	0.672
RV/LV	1.321 (1.230–1.418)	<0.001	1.128 (0.971–1.310)	0.117
hs-cTnI	5.066 (2.877–8.920)	<0.001	2.690 (1.185–6.106)	0.018
BNP	1.003 (1.002–1.004)	<0.001	1.001 (0.999–1.002)	0.423
D-dimer	1.133 (1.010–1.272)	0.033	1.255 (1.092–1.443)	0.001
BLa	1.861 (1.604–2.160)	<0.001	1.655 (1.240–2.209)	0.001
sPESI strata	3.764 (2.000–7.084)	<0.001	0.478 (0.163–1.399)	0.178

SBP, systolic blood pressure; SpO_2_, peripheral oxygen saturation; PAOI, pulmonary artery obstruction index; RV/LV, right/left ventricular diameter ratio; hs-cTnI, high-sensitivity cardiac troponin I; BNP, brain natriuretic peptide; BLa, blood lactate; sPESI, simplified Pulmonary Embolism Severity Index; HR, hazard ratio; CI, confidence interval.

### 3.6 Correlation analysis between CT imaging parameters and serum biomarkers

**Figure 8:**
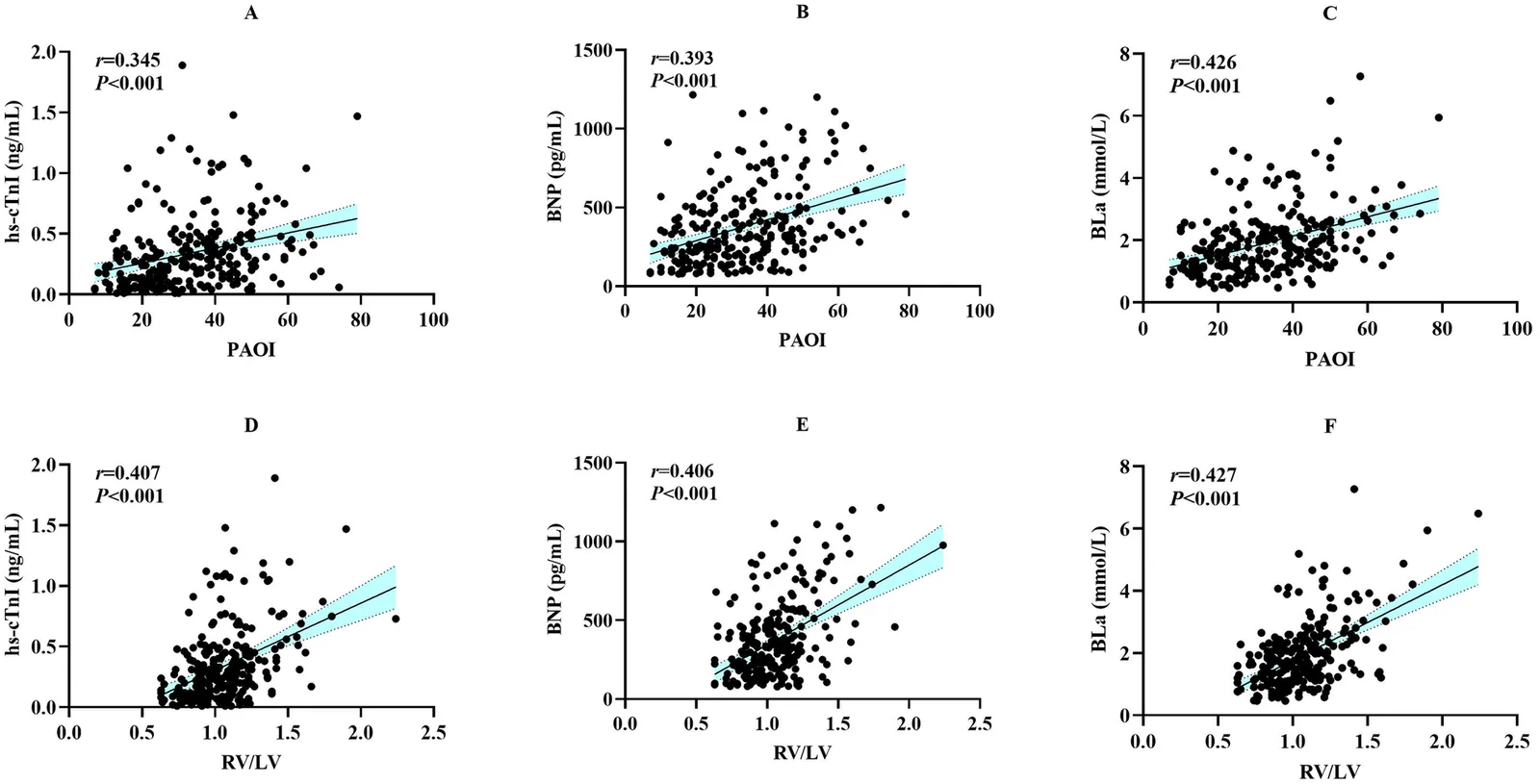
Correlation scatter plots between CT imaging parameters and serum biomarkers. **(A)** Scatter plot of PAOI and hs-cTnI; **(B)** Scatter plot of PAOI and BNP; **(C)** Scatter plot of PAOI and BLa; **(D)** Scatter plot of RV/LV and hs-cTnI; E. Scatter plot of RV/LV and BNP; F. Scatter plot of RV/LV and BLa. CT, computed tomography; PAOI, pulmonary artery obstruction index; hs-cTnI, high-sensitivity cardiac troponin I; BNP, brain natriuretic peptide; BLa, blood lactate; RV/LV, right/left ventricular diameter ratio.

According to Spearman correlation analysis, PAOI was positively correlated with hs-cTnI, BNP, and BLa (*r* = 0.345, 0.393, and 0.426, respectively; all *P* < 0.001). Similarly, RV/LV showed positive correlations with hs-cTnI, BNP, and BLa (*r* = 0.407, 0.406, and 0.427, respectively; all *P* < 0.001; Figure 8).

### 3.7 Comparison of 30-day all-cause mortality, CTPA imaging parameters, and serum biomarkers between risk strata

**Figure 9:**
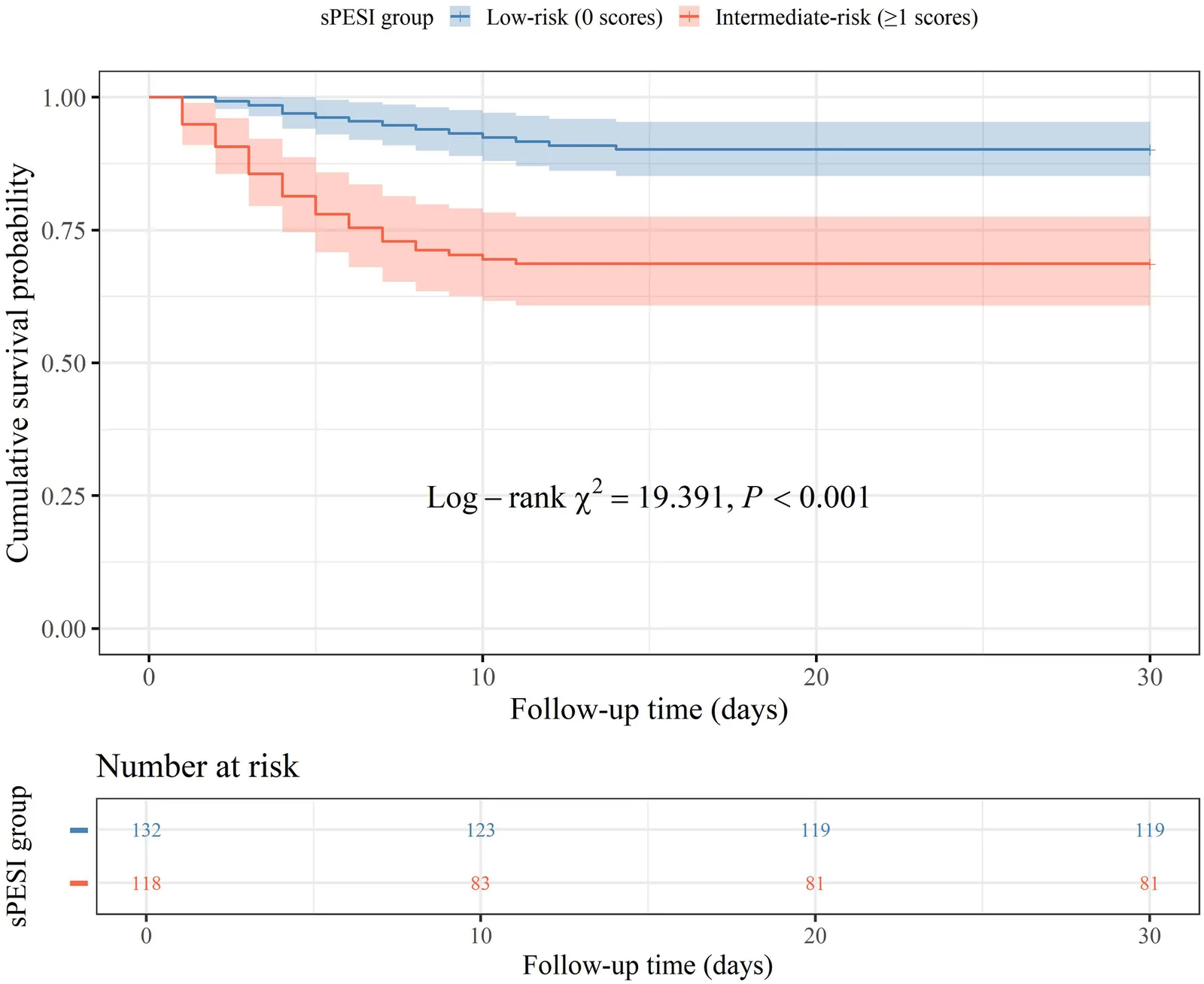
Kaplan–meier survival curves for 30-day all-cause mortality in patients stratified by sPESI risk level. sPESI, simplified Pulmonary Embolism Severity Index.

The intermediate-risk group exhibited significantly higher 30-day all-cause mortality, PAOI, RV/LV, BNP, and BLa levels compared with the low-risk group (all *P* < 0.05). No significant between-group differences were detected in PA/AO, hs-cTnI, or D-dimer (all *P* > 0.05; Table 6). The Kaplan–Meier curve (Figure 9) confirmed that the 30-day cumulative survival rate was lower in the intermediate-risk group compared with the low-risk group (Log-rank *χ*^2^ = 19.391, *P* < 0.001).

**Table 6:** Comparison of CTPA imaging parameters and serum biomarkers between risk strata.

Variable	Intermediate-risk group (*n* = 118)	Low-risk group (*n* = 132)	*χ* ^2^ */Z*	*P*
30-day all-cause mortality	37 (31.36)	13 (9.85)	18.012	<0.001
PAOI	36.00 (23.00, 47.00)	29.50 (21.00, 39.00)	2.450	0.014
RV/LV	1.06 (0.92, 1.25)	1.01 (0.90, 1.15)	2.541	0.011
PA/AO	0.90 (0.80, 1.00)	0.88 (0.78, 0.97)	1.671	0.095
hs-cTnI (ng/mL)	0.31 (0.13, 0.48)	0.24 (0.11, 0.40)	1.383	0.167
BNP (pg/mL)	357.00 (214.00, 571.00)	302.50 (182.00, 457.50)	2.141	0.032
D-dimer (mg/L)	3.31 (1.63, 5.22)	2.92 (1.42, 4.57)	1.758	0.079
BLa (mmol/L)	2.00 (1.23, 2.61)	1.57 (1.12, 2.13)	2.919	0.004

CTPA, computed tomography pulmonary angiography; PAOI, pulmonary artery obstruction index; RV/LV, right/left ventricular diameter ratio; PA/AO, main pulmonary artery/ascending aorta diameter ratio; hs-cTnI, high-sensitivity cardiac troponin I; BNP, brain natriuretic peptide; BLa, blood lactate.

## 4 Discussion

In this study, we constructed a nomogram incorporating syncope, SBP <100 mmHg, RV/LV, BNP, and BLa to predict 30-day all-cause mortality in non-high-risk APE patients. This model demonstrated excellent predictive performance and clinical utility, significantly surpassing the traditional sPESI score. The NRI and IDI analyses confirmed its incremental value in risk reclassification and discrimination. These findings suggest that integrating CTPA-derived RV function indices and serum biomarkers of tissue perfusion with bedside sPESI parameters provides a more comprehensive capture of the pathophysiological mechanisms driving adverse outcomes, leading to more precise mortality risk assessment.

Among the independent predictors identified by Firth penalized likelihood logistic regression, syncope and SBP <100 mmHg exhibited the strongest effects. Syncope, a common clinical manifestation of APE, is thought to result from massive thromboembolism causing a sudden increase in RV afterload, reduced LV filling, decreased cardiac output, and transient cerebral hypoperfusion. Its occurrence often indicates a significant thrombus burden and a critically poised hemodynamic reserve, where compensatory mechanisms may be nearing decompensation even if blood pressure is within normal limits at presentation (16, 17). A retrospective study of pulmonary embolism patients found syncope in 12.4% of cases, associated with an approximately 4.2-fold increased risk of 30-day all-cause mortality (16). Our results reinforce that syncope holds independent prognostic warning value even in non-high-risk APE patients, warranting closer monitoring for those presenting with syncope. Similarly, while SBP <100 mmHg does not meet the criteria for high-risk shock or persistent hypotension, it signifies an early stage of hemodynamic compromise. APE-induced RV afterload increase and septal shift impair LV filling, and reduced cardiac output initially manifests as a mild SBP decrease (18). An SBP <100 mmHg suggests that the RV might be entering a pre-decompensation phase, significantly worsening prognosis (19). This aligns with the rationale of the ESC guidelines for including SBP <100 mmHg in the sPESI score (20), and underscores it as a crucial warning sign even within the non-high-risk cohort.

Regarding CT imaging parameters, RV/LV was an independent predictor of 30-day all-cause mortality. RV/LV, a core CTPA index of RV dysfunction, reflects RV dilation in response to increased afterload and serves as direct imaging evidence of RV impairment (21). A meta-analysis reported that RV/LV ≥1.0 was associated with an approximately 2.5-fold increased risk of 30-day all-cause mortality in APE patients (22, 23). Our analysis, treating RV/LV as a continuous variable, revealed an *OR* of 1.285 per 0.1 unit increase, finely characterizing the dose-response relationship between RV dilation and mortality risk. This suggests that clinicians should consider continuous changes in RV/LV, rather than solely relying on the binary cut-off of ≥1.0. Furthermore, Spearman correlation analyses showed that RV/LV was positively correlated with hs-cTnI, BNP, and BLa, suggesting that RV dilation, myocardial injury, ventricular stretch, and tissue hypoperfusion are interconnected pathophysiological components that collectively contribute to poor outcomes.

Among serum biomarkers, BNP and BLa were identified as independent predictors of 30-day all-cause mortality. BNP, secreted by ventricular myocytes in response to pressure or volume overload, is a sensitive marker of ventricular wall stress. In APE, the acute increase in RV afterload raises ventricular wall tension, stimulating BNP release (6). The significantly higher BNP levels in the death group compared to the survival group suggest a strong link between ventricular wall stretch and prognosis. Similarly, the intermediate-risk sPESI group had higher BNP levels than the low-risk group, supporting BNP's complementary value in risk stratification. Elevated BLa directly reflects tissue hypoperfusion and cellular hypoxia. The mechanisms of BLa elevation in APE involve reduced cardiac output from RV dysfunction and increased peripheral anaerobic metabolism (24). The significantly higher BLa in the death group, and in the intermediate-risk sPESI group, indicates that subclinical tissue hypoperfusion can exist and correlate with short-term prognosis even in non-high-risk patients. Moreover, unlike BNP (reflecting ventricular stretch) and hs-cTnI (reflecting myocardial injury), BLa represents an integrated measure of systemic tissue perfusion, potentially offering a more comprehensive information dimension, which might explain its stronger effect size in the Firth regression compared to hs-cTnI.

The independent predictors identified by Firth penalized likelihood logistic regression and multivariate Cox regression were not entirely concordant. Variables like age >80 years, pulse ≥110 bpm, hs-cTnI, and D-dimer were independent risk factors in the Cox model but did not reach statistical significance in the Firth regression. This likely stems from methodological differences. Firth regression, by introducing a Jeffreys prior penalty, effectively addresses small-sample bias and complete separation in rare events data, but under this penalization, predictors with smaller effect sizes or collinearity with others may be shrunk towards zero, retaining only the strongest, most robust signals. Cox regression, conversely, models the full survival time data across the entire cohort and may better capture risk signals whose effects are persistent over the follow-up period but less pronounced at a single time point (25). From a biological perspective, patients >80 years are more prone to hemodynamic instability and ventricular stretch, with much of their prognostic information potentially captured by variables like SBP <100 mmHg, BNP, and BLa, which more directly reflect circulation and ventricular load, thereby partially explaining the effect of age (23). Tachycardia is a compensatory response to RV dysfunction, and its effect may be more precisely quantified by RV/LV and BNP (26). Hs-cTnI, a highly sensitive marker of myocardial injury typically resulting from RV pressure overload (27), was significantly higher in the death group but did not enter the Firth model. Possible reasons include the exclusion of high-risk patients (where hs-cTnI's predictive value is more prominent (28)) and its limited discriminative power within the non-high-risk cohort, as suggested by the non-significant difference between sPESI strata. Furthermore, its moderate correlation with RV/LV suggests that its effect might be partially supplanted by RV/LV. Therefore, the prognostic value of hs-cTnI in this population may only become apparent when combined with other markers. D-dimer presents a more complex picture; its concentration depends not only on thrombus burden but also on age, renal function, fibrinolytic activity, and metabolic differences, leading to heterogeneous prognostic value across studies (29–31). Under Firth regression's strict penalization, D-dimer's relatively weaker signal was excluded, whereas in Cox regression leveraging survival time, its role as a global indicator of coagulation-fibrinolysis activation yielded a detectable, albeit modest, persistent prognostic effect. The discrepancies between the two regression methods are a natural consequence of both their methodological properties and the biological nature of the variables. They are not contradictory but complementary: Firth regression yields a parsimonious clinical prediction tool with the strongest signals, while Cox regression captures variables with temporally persistent effects on survival time. Overall, syncope, SBP <100 mmHg, and BLa demonstrated robust independent predictive ability in both models and can serve as core warning indicators, while the value of age, pulse, hs-cTnI, D-dimer, and RV/LV varies by model and patient characteristics, warranting integrated, dynamic interpretation in clinical practice.

PAOI was significantly higher in the death group vs. the survival group but did not reach statistical significance in the multivariable Firth regression; moreover, after adjustment for other variables, PAOI showed a weak negative association with mortality risk. Spearman correlation analysis demonstrated that PAOI was positively correlated with BNP and BLa, both of which were independent predictors. When these variables were included simultaneously in the model, BNP and BLa accounted for the majority of the explained variance, and the prognostic information of PAOI was supplanted by these more direct functional indicators of ventricular wall stretch and tissue hypoperfusion, with the residual effect manifesting as dilution and reversal due to collinearity (32, 33). From a pathophysiological perspective, PAOI reflects anatomical thrombus burden, whereas BNP and BLa reflect its functional consequences (34). Some patients with high PAOI may have favorable prognosis due to adequate compensation, while others with low PAOI may have poor prognosis due to limited cardiopulmonary reserve.

Our multivariate Cox regression analysis revealed that after adjusting for multiple factors, sPESI strata were no longer an independent predictor of 30-day all-cause mortality. This suggests that the prognostic information encompassed by sPESI strata can be largely explained by the specific clinical and biochemical indicators in our model. Interaction analysis showed no statistically significant interaction between sPESI strata and RV/LV or BLa, meaning that sPESI strata do not modify the direction or strength of the association between RV/LV or BLa and mortality risk. This finding has important clinical implications. First, the prognostic information from CTPA-derived RV function indices and serum tissue perfusion markers appears independent of sPESI strata; they are not simple substitutes but capture complementary pathophysiological information. Second, this underscores the value of a combined assessment strategy. Regardless of whether a patient is classified as low- or intermediate-risk by sPESI, elevated RV/LV and BLa should prompt clinical attention. Conversely, even intermediate-risk patients with normal RV/LV and BLa might have lower-than-expected short-term mortality risk.

The sPESI stratification results showed significantly higher 30-day all-cause mortality in the intermediate-risk group, and the Kaplan–Meier curves confirmed a significant difference in cumulative survival, indicating that sPESI retains good prognostic discrimination within the non-high-risk cohort. This also indirectly validates the rationale of incorporating sPESI information into our nomogram. The intermediate-risk group also exhibited higher PAOI, RV/LV, BNP, and BLa than the low-risk group, suggesting greater thrombus burden and more pronounced RV dysfunction, ventricular stretch, and tissue hypoperfusion, forming the pathophysiological basis for increased adverse prognosis risk. PA/AO, hs-cTnI, and D-dimer did not differ significantly between sPESI strata. PA/AO, mainly reflecting main pulmonary artery dilation, may have limited discriminative ability in acute events due to influences from body habitus, scan phase, and chronic vascular remodeling (35). The non-significant difference in hs-cTnI between groups, coupled with its failure to emerge as an independent predictor in Firth regression, further supports that after excluding high-risk patients, the prognostic discrimination of cardiac troponin tends to weaken, and its prognostic significance in this population may be overlapped by RV function indices.

This study has several limitations. First, it is a single-center retrospective case-control study with a limited sample size, and internal validation via bootstrap resampling was not supplemented by external validation using independent multi-center cohorts or public ICU databases (e.g., MIMIC-IV, eICU). Therefore, there is a potential risk of overfitting, and the generalizability and robustness of the model across other centers and populations remain to be confirmed. Second, using 30-day all-cause mortality as the endpoint without distinguishing causes of death may affect specificity. Third, laboratory markers lacked standardized time points and dynamic trend data. Fourth, regarding early risk stratification, we only compared our nomogram with the sPESI score. Future comparisons with other bedside tools, such as qSOFA, which has shown value in early pulmonary embolism mortality risk stratification (36), would provide a more comprehensive assessment of the nomogram's strengths and weaknesses relative to existing tools. Fifth, our biomarker selection focused on myocardial injury, ventricular stretch, and hypoperfusion. Recent studies suggest that systemic inflammation biomarkers (e.g., neutrophil/lymphocyte ratio, platelet/lymphocyte ratio) could serve as useful adjuncts for risk assessment (37), supporting the rationale for integrating multi-dimensional biomarkers into multimodal prediction models and providing avenues for future optimization. Future research should prioritize designing multi-center, prospective cohort studies with larger sample sizes for independent external validation. Incorporating qSOFA and inflammatory markers could systematically evaluate the nomogram's generalizability and clinical utility. Further refinement could involve integrating multimodal imaging and dynamic biomarker data, with prospective interventional studies needed to confirm clinical net benefit.

In conclusion, this study successfully constructed a nomogram incorporating syncope, SBP <100 mmHg, RV/LV, BNP, and BLa to predict 30-day all-cause mortality in non-high-risk APE patients. The model demonstrates good discrimination, calibration, and clinical net benefit, with predictive performance significantly superior to the sPESI score. Positive correlations exist between CT imaging parameters and serum biomarkers, with significant differences across sPESI strata. The findings suggest that combined assessment provides independent and complementary prognostic information beyond the sPESI score, potentially overcoming the limitations of traditional stratification tools and offering new evidence for individualized, precise risk stratification and clinical decision-making.

## Data Availability

The raw data supporting the conclusions of this article will be made available by the authors, without undue reservation.
